# Revisiting primary neural leprosy: Clinical, serological, molecular, and neurophysiological aspects

**DOI:** 10.1371/journal.pntd.0006086

**Published:** 2017-11-27

**Authors:** Diogo Fernandes dos Santos, Matheus Rocha Mendonça, Douglas Eulálio Antunes, Elaine Fávaro Pípi Sabino, Raquel Campos Pereira, Luiz Ricardo Goulart, Isabela Maria Bernardes Goulart

**Affiliations:** 1 National Reference Center for Sanitary Dermatology and Leprosy, Clinics’ Hospital, School of Medicine, Federal University of Uberlândia (UFU), Uberlândia, MG, Brazil; 2 Postgraduate Program in Health Sciences, School of Medicine, Federal University of Uberlândia (UFU), Uberlândia, MG, Brazil; 3 Institute of Genetics and Biochemistry, Federal University of Uberlândia (UFU), Uberlândia, MG, Brazil; University of Tennessee, UNITED STATES

## Abstract

**Background:**

Leprosy neuropathy is considered the most common peripheral neuropathy of infectious etiology worldwide, representing a public health problem. Clinical diagnosis of primary neural leprosy (PNL) is challenging, since no skin lesions are found and the slit skin smear bacilloscopy is negative. However, there are still controversial concepts regarding the primary-neural versus pure-neural leprosy definition, which will be explored by using multiple clinical-laboratory analyses in this study.

**Methodology/Principal findings:**

Seventy patients diagnosed with primary neural leprosy from 2014 to 2016 underwent clinical, laboratorial and neurophysiological evaluation. All patients presented an asymmetric neural impairment, with nerve thickening in 58.6%. Electroneuromyography showed a pattern of mononeuropathy in 51.4%. Positivity for ELISA anti-PGL1 was 52.9%, while the qPCR of slit skin smear was 78.6%. The qPCR of nerve biopsies was positive in 60.8%. Patients with multiple mononeuropathy patterns showed lower levels of anti-PGL-1 (p = 0.0006), and higher frequency of neural thickening (p = 0.0008) and sensory symptoms (p = 0.01) than those with mononeuropathy.

**Conclusions/Significance:**

PNL is not a synonym of pure neural leprosy, as this condition may include a generalized immune response and also a skin involvement, documented by molecular findings. Immunological, molecular, and neurophysiological tools must be implemented for diagnosing primary neural leprosy to achieve effective treatment and reduction of its resultant disabilities that still represent a public health problem in several developing nations. Finally, we propose a algorithm and recommendations for the diagnosis of primary neural leprosy based on the combination of the three clinical-laboratorial tools.

## Author summary

The long incubation period, insidious symptoms and signs of leprosy produce difficulties in its diagnosis and correct clinical classification, especially in its primary neural form characterized by negative bacilloscopy and lack of cutaneous lesions. Despite significant progress in leprosy control in recent years, early identification of cases remains one of the primary goals of leprosy control programs. In addition, the failure of current therapeutic schemes on the incidence of leprosy shows that elimination of this disease, a public health problem, depends on incisive action to interrupt its transmission chain. Therefore, efforts must be focused on improving the leprosy diagnosis. However, because it is a primarily neural disease, the use of neurophysiological, serological and molecular methods in this group of patients may contribute not only to early diagnosis, but also to a correct classification and treatment, preventing sequelae and controlling outbreaks of infection. Therefore, in order to improve the leprosy elimination program in many countries, we propose the implementation of a combination of specific tools for detecting *M*. *leprae* and its neural impairment, which may further break down the transmission chain, enabling a more effective control of the disease.

## Introduction

Leprosy is a chronic infectious disease caused by the *Mycobacterium leprae*, an alcohol- and acid-resistant obligatory intracellular bacillus with predilection to infect peripheral nerves and skin. Its clinical forms are defined by the host immune response and bacillary load, resulting in a wide clinical spectrum [1,2,3].

Leprosy neuropathy is considered the most common peripheral neuropathy of infectious etiology worldwide, representing a public health problem, mainly due to its incapacitating potential and strong social discrimination and stigma [4].

Leprosy is classified into five clinical forms according to the Ridley-Jopling, proposed in 1966, which is based on skin lesion histopathology and bacterial load. According to this classification, cases with cellular immune response mediated by T lymphocytes are classified as tuberculoid (TT), while anergic patients with humoral response are considered to be suffering from lepromatous leprosy (LL). Patients between these two extremes are defined as borderline, presenting intermediate immune responses. For operational purposes aiming to achieve proper treatment regimens, patients are divided into paucibacillary (PB) or multibacillary (MB) forms, according to their bacilloscopic index (BI), the number of skin lesions and affected nerves [5,6,7].

The *M*. *leprae* bacillus causes multiple mononeuropathy, which may result in autonomic, sensory and motor dysfunction. Histopathologically, there are myelinic and axonal dysfunctions, followed by substitution of the nervous tissue by connective tissue and fibrosis. The peripheral neural impairment includes nerve trunks as well as distal cutaneous branches. Sensory symptoms often correspond to the initial and most common complaints, always with an asymmetrical impairment. Mononeuropathy, multiple mononeuropathy and confluent mononeuropathy are the most common clinical presentations [8,9,10].

Primary neural leprosy (PNL), also known as pure neural or neuritic leprosy form, was initially described by the Indian classification of 1955 [11]; since then it became a challenging clinical diagnosis. This clinical form is characterized by no skin lesions and negative slit skin smear bacilloscopy. Therefore, diagnosis is mainly based on supplementary tests, such as electroneuromyography, nerve biopsy, serology and molecular analyses [12,13,14,15,16,17]. However, most of the leprosy reference services do not have access to advanced diagnostic methods; therefore, diagnosis is based mainly on clinical manifestations, such as the absence of skin lesions and negative skin smears bacilloscopy. But the scarcity of symptoms at the disease onset has commonly led to diagnostic errors and under diagnosis of the neural clinical form. For this reason, several laboratory tools are required for the investigation of this neuropathy, enabling not only early diagnosis, but contributing to the prevention of incapacities [12,18,19].

Our aim was to characterize the clinical, neurophysiological, serological, and molecular patterns of patients with PNL diagnosis, which led us to propose the combination of these tools to achieve an early diagnosis and to better control the disease.

## Patients and methods

### Ethics statement

The Ethics Committee of the Federal University of Uberlandia approved the study (CAAE: 48293215.7.0000.5152). Written informed consent was obtained from all participants. Some participants were minors and their parents provided written consent on behalf of them.

### Subjects

Seventy individuals with diagnosis of PNL were recruited among 317 new cases from July 2014 to July 2016 treated at the National Reference Center of Sanitary Dermatology and Leprosy (CREDSH), Uberlandia, MG, Brazil.

The PNL diagnosis fulfilled the following criteria: clinical evidence of peripheral neuropathy associated with the absence of skin lesions and negative slit skin smear bacilloscopy [12]. Patients who showed other possible etiologies of peripheral neuropathies during diagnosis were excluded, namely those with: chronic alcoholism, diabetes mellitus, thyroid disease and/or other hormonal dysfunctions, malnutrition, hereditary neuropathy, hepatitis B or C, HIV, rheumatic and/or autoimmune diseases.

### Clinical characterization

Epidemiological (age, gender, previous contact with leprosy cases) and clinical (initial symptoms, sensory impairment modalities, presence of muscle weakness and amyotrophy, neural thickening, deep reflexes evaluation) data were examined. The level of functional disability was evaluated, according to the recommended protocol of the Ministry of Health [20], which evaluates the neural function integrity and the degree of physical disability during diagnosis, through muscle strength, and sensoriality tests of the hands and feet. In relation to the grade 2 disability caused by leprosy, observations were based on the presence of visible deficiencies, such as claws (clawing of digits), bone resorption, muscular atrophy, contractures and wounds. All patients underwent a rigorous dermatoneurological evaluation by expert professionals.

### Laboratory analyses

Slit skin smears–Slit skin smears from six sites wasperformed: the two ear lobes, bothelbows and the two knees, as well as from skin and/or nerve biopsy samples.The sample collection was preceded by topical application of cream containing lidocaine (7%) and tetracaine (7%) atall sites, awaiting the anesthetic effect for one hour. The vial with phosphate buffer is sterile and all collected material is immediately sent to the laboratory of molecular pathology and biotechnology. The tubes are always individually processed and compared with two negative controls to ensure that the sample is not contaminated.

Bacilloscopy–Bacilloscopic analyses were performed on slit skin smears from six sites (two ear lobes, two elbows, two knees), and skin and/or nerve biopsy samples.

ELISA anti-PGL-1 serology–Serum IgM antibodies were detected by enzyme-linked immunosorbent assay (ELISA) performed against the purified native PGL-I from the *Mycobacterium leprae* cell wall, as described elsewhere [21].

DNA Extraction and Real Time Quantitative Polymerase Chain Reaction (Real Time PCR)–DNA extraction from dermal smear samples, nerve biopsies, and superjacent skin was performed. To detect *M*. *leprae* DNA, a previously described quantitative real-time PCR (qPCR) primer/probe assay targeting the *M*. *leprae* species-specific genomic element of dispersed repeats (RLEP) was performed in the real-time PCR system ABI 7300 (Applied Biosystems, Foster City, CA, USA) [11,22,23,24]. In the laboratory the vials are always individually processed and compared with two negative controls to ensure that the sample is not contaminated.

### Electroneuromyography

Electroneuromyographic studies were performed using a MEB 4200K (NIHON-KODHEN) electroneuromyographer. For the sensory conduction analysis, the median, ulnar, dorsal hand cutaneous, radial, lateral antebrachial cutaneous, median antebrachial cutaneous, sural, and fibular superficial bilaterally nerves were examined. For the motor conduction analysis, the median, ulnar, common fibular, and tibial bilaterally nerves were examined, supplemented by techniques for focal impairment identification at compression sites often affected in leprosy neuropathy, such as median nerve at the wrist, ulnar nerve at the elbow, fibular nerve at the fibular head and tibial nerve at the ankle.

### Skin biopsy

According to the PNL concept, none of the patients presented skin lesions. For this reason, biopsies were performed on the small elbow skin fragment, which is the coldest region with a possible intradermal impairment, and a site often attacked in leprosy neuropathy, even without an evident local skin lesion.

### Nerve biopsy

Nerves that underwent biopsy were selected according to the patient’s clinical condition, and included exclusively sensory nerves that presented sensory changes and/or thickening, and also one of the following electrophysiological changes in the sensory conduction analysis: absence of response on both sides; unilateral absence of response; bilaterally decreased amplitude of the sensory nerve action action potential (SAP), considering reference values; and over 50% decrease in the amplitude of the SAP, compared with the contralateral side. During the biopsy, the nerve was isolated and completely transected. All patients signed a specific informed consent form referring to this process. During the procedure, a skin biopsy of the area superjacent to the corresponding territory of the nerve also underwent a biopsy procedure. The biopsied nerve and skin were processed and studied according to routine standard procedures. Formalin-fixed paraffin-embedded were cut longitudinally and transversely at 5 μ thickness and stained with hematoxylin and eosin stain. In addition special stains like Masson Trichome to assess fibrosis. Fite-Faraco stain was performed for bacilli identification.

### Statistical analysis

Continuous and dichotomous variables were applied to evaluate differences of clinical and laboratory factors among groups. The Shapiro Wilk test was applied to verify data normality prior to applying parametric or non-parametric analyses. The Wilcoxon-Mann-Whitney U Test was carried out to compare differences between independent groups when the dependent variables were not normally distributed. The Binomial Test was applied to evaluate dichotomous variables. The statistical software used was GraphPad Prism version 7 (La Jolla, CA, USA), and all tests presenting a probability below 5% were considered significant.

## Results

Seventy patients diagnosed with PNL, from 2014 to 2016, were included in this study and 44.3% (31/70) of these were household contacts of patients with a previous leprosy diagnosis. The average age was 42.9 (±17.3) years, and 52.9% (37/70) were male. Slit skin smear bacilloscopy of the six sites (ear lobes, elbows, and knees) was negative in all patients, who also did not present any skin lesions compatible with leprosy.

Among patients, 61.4% (43/70) were clearly symptomatic. All assymptomatic patients (27/70) were household contacts of leprosy patients, who were annually assisted by epidemiological surveillance, throughout a 7-year period, and presented some electroneuromyographic abnormality, reinforcing the need for a detailed interview and an active search for neurological signs and symptoms in this high-risk group.

All symptomatic patients presented an asymmetric neural impairment, with a predominance of sensory symptoms, particularly hypoesthesia, paresthesia and pain, evidenced by thermal, painful, and/or tactile impairment, in addition to an intradermic sensory involvement in 69.8% (30/43). Deep reflexes and vibration sensation changes were present in only 8.6% (6/70) of the cases, while 30% (21/70) complained of muscular weakness and/or amyotrophy, besides the sensory symptoms.

Neural thickening of one or more nerves was observed in 58.6% (41/70) of the patients, of which 75.6% (31/41) presented focal myelin impairments in the electroneuromyographic evaluation. As to the disability degree during diagnosis, 20% (14/70) presented grade 2 disability, mostly evidenced by the presence of muscular weakness and amyotrophy.

In relation to the electroneuromyographic evaluation, an average of only 2.3 altered nerves per patient was observed. The most frequently affected nerves were the ulnar in the elbow segment (34.4%; 56/163), common fibular in the fibula head segment (30.2%; 33/163), followed by the sensory ulnar (12.8%; 20/163), superficial fibular (10.4; 17/163), and sural nerve (6.1%; 10/163) (Table 1).

**Table 1:** Distribution of the most affected peripheral nerves in patients with primary neural leprosy diagnosis.

Affected Nerve	n	%
Ulnar (Elbow)	56	34.4
Common fibular	33	20.2
Sensory ulnar	20	12.8
Superficial fibular	17	10.4
Sural	10	6.1
Motor median	8	4.9
Superficial radial	6	3.7
Sensory median	4	2.4
Tibial	4	2.4
Median antebrachial cutaneous	2	1.2
Lateral antebrachial cutaneous	2	1.2
Deep fibular	1	0.6
**Total nerves**	163	100

As to the neurophysiological pattern observed in electroneuromyography, 51.4% (36/70) presented only one altered nerve (mononeuropathy), while 48.6% (34/70) presented two or more affected nerves (asymmetrical multiple mononeuropathy). The electromyographic pattern and its distribution are detailed in Table 2.

**Table 2:** Distribution of the electroneuromyographic pattern in patients with primary neural leprosy diagnosis.

Electroneuromyographic pattern	n	%
Focal demyelinating mononeuropathy	19	27.1
Sensory axonal mononeuropathy	17	24.3
Asymmetrical sensory and motor demyelinating neuropathy	21	30.0
Asymmetrical sensory and motor axonal neuropathy with focal slowing of conduction velocity	13	18.6
Total	70	100

The ELISA anti-PGL1 IgM serology was positive in 52.9% (37/70) of the cases. The qPCR test in slit skin smears was positive in 78.6% (55/70) of the cases, which was much higher than bacilloscopy (negative in all cases). It should be emphasized that only 21.4% (15/70) of the neural cases showed negative qPCR in slit skin smears.

In relation to the skin biopsy in the elbow area (routine lab services in all cases with absence of suspected dermatological lesions), a positivity of 30% (21/70) for *M leprae* DNA was observed, whereas only one case (1.4%) showed positive bacilloscopy in the biopsy of this area.

According to clinical data and the electroneuromyography results, 57.1% (40/70) of patients demonstrated at least one eligible nerve for biopsy, but only 70% (28/40) of those were submitted to this process. The most frequent nerve submitted to biopsy was the sensory ulnar—dorsal cutaneous of the hand (82.1%; 23/28), followed by superficial fibular (10.7%; 3/28), sural (3.6%; 1/28), and deep fibular (3.6%; 1/28). Only 13.8% (4/28) of the nerves that underwent biopsy presented some histopathological alterations, suggestive of leprosy, such as endoneural or epineural infiltrate, presence of fibrosis, perineural thickening or presence of endoneural granuloma. Only one case (3.5%) presented positive bacilloscopy in the peripheral nerve biopsy. On the other hand, qPCR of nerve biopsies was positive in 60.8% (17/28) of the cases. The qPCR of the superjacent skin area was positive in only 10.7% (3/28) of the nerve biopsies, with negative bacilloscopy in all samples.

To achieve a better understanding of the disease phenotype and clinical progression, patients were divided into two groups: one composed of individuals presenting electroneuromyographic pattern of mononeuropathy (51.4%; 36/70), and the other of those presenting a multiple mononeuropathy pattern (48.6%; 34/70) (Table 3).

**Table 3:** Distribution of patients with primary neural leprosy according to the electroneuromyographic pattern, and comparisons of proportions.

Parameters	Mononeuropathy	Multiple Mononeuropathy	*p* value
**ELISA antiPGL-1 index**	1.54 ±1.1	0.82 ±0.63	p = 0.0006
**Neural thickening**	33.3% (12/36)	73.6% (25/34)	p = 0.0008
**Sensory symptoms**	47.2% (17/36)	76.5% (26/34)	p = 0.0120
**Muscular weakness**	22.2% (8/36)	38.3% (13/34)	p = 0.1440
**qPCR peripheral blood**	38.9% (14/36)	23.5% (8/34)	p = 0.1665
**qPCR slit skin smear**	88.9% (32/36)	67.65% (23/34)	p = 0.0304

qPCR = Real-Time Quantitative Polymerase Chain Reaction. ELISA = enzyme-linked immunosorbent assay

For the group of patients with multiple mononeuropathy patterns, lower levels of ELISA antiPGL-1 were observed (p = 0.0006), as well as higher neural thickening frequency (p = 0.0008), and sensory symptoms (p = 0.01). A higher motor symptom incidence, although not significant, was also found in this group (p = 0.1440), reinforcing the greater severity of neural damage. The positivity of the qPCR in slit skin smears was smaller in this group (p = 0.03) (Table 3).

Table 4 details a new analysis of the clinical and laboratory profile of two new groups of PNL patients, defined by their seropositivity to the ELISA anti-PGL1 test. The seronegative group presented a larger number of impaired nerves (p = 0.003), higher proportion of neural thickening (p = 0.001), sensory symptoms (p<0.0001) and motor symptoms (p = 0.001). Lower positivity of qPCR in the peripheral blood, slit skin smear and skin biopsy were observed, reinforcing that the bacillary load in this group was lower, although not statistically different (Table 4).

**Table 4:** Distribution of patients with primary neural leprosy according to ELISA anti-PGL1 seropositivity and comparisons of proportions.

Parameters	ELISA anti-PGL1 Negative	ELISA anti-PGL1 Positive	*p* value
**ELISA index**	0.52 ±0.32	1.79 ±1.0	p<0.0001
**Number of altered nerves**	3.18 ±4.1	1.43 ±0,86	p = 0.0033
**Neural thickening**	78.8% (26/33)	40.5% (15/37)	p = 0.0012
**Sensory symptoms**	90.1% (30/33)	35.1% (13/37)	p< 0.0001
**Muscular weakness**	48.5% (16/33)	13.5% (5/37)	p = 0.0014
**Peripheral blood qPCR**	27.3% (9/33)	35.1% (13/37)	p = 0.48
**Intradermic smear qPCR**	72.7% (24/33)	83.8% (31/37)	p = 0.26
**Skin biopsy qPCR**	21.2% (7/33)	37.8% (14/37)	p = 0.12

qPCR = Real-Time Quantitative Polymerase Chain Reaction. ELISA = enzyme-linked immunosorbent assay

The combined evaluation of all diagnostic tools (Table 5) demonstrated a highly variable presentation, with 17.1% of patients being positive only in slit skin smear qPCR and 8.6% being negative in all tests, reinforcing the importance of peripheral nerve biopsy in some cases.

**Table 5:** Combination of diagnostic tools for primary neural leprosy diagnosis, considering suspicious clinical cases with positive electroneuromyography.

ELISA anti-PGL-1	Peripheral blood qPCR	Slit skin smears qPCR	Skin biopsy qPCR	Number of Patients (%)
╋	+	+	+	3 (4.3)
			-	8 (11.4)
		-	+	-
			-	2 (2.9)
	-	+	+	9 (12.9)
			-	11 (15.7)
		-	+	2 (2.9)
			-	2 (2.9)
━	+	+	+	1 (1.4)
			-	6 (8.6)
		-	+	-
			-	2 (2.9)
	-	+	+	5 (7.1)
			-	12 (17.1)
		-	+	1 (1.4)
			-	6 (8.6)

qPCR = Real-Time Quantitative Polymerase Chain Reaction. ELISA = enzyme-linked immunosorbent assay

## Discussion

The present study characterizes for the first time the clinical, serological, molecular, and neurophysiological aspects of 70 patients with PNL diagnosis, assisted in a leprosy national reference center in Brazil from 2014 to 2016, evidencing the importance of using multiple analytical tools for proper diagnosis of this neuritic leprosy form.

The prevalence of this clinical form was 22.1%, considering the 317 diagnosed cases during this period, contrasting with the very low prevalence (5.5% to 17.1%) found elsewhere [12,13,14,16], evidencing the difficulty in diagnosing this leprosy form. Probably, this difficulty is due to the absence of serological, molecular, and neurophysiological diagnostic tools, thus accounting for the under diagnosed of many cases, leading to the late diagnosis of this disease. In the present study, we have observed not only a high proportion of neural cases, but also a premature recognition of this clinical form, obtained by a combination of diagnostic tools and an active search of cases through the evaluation of household contacts.

Interestingly, 38.6% of the patients were diagnosed during an epidemiological surveillance and follow-up of household contacts of leprosy patients, supporting the need for control measures and early disease recognition in this high risk population. It is estimated that household contacts of multibacillary patients may present a relative risk of developing leprosy 5 to 10 times higher than the general population [25,26,27].

The clinical presentation pattern was defined in all patients, and the asymmetric impairment of symptoms was observed in all cases. The sensory symptoms were the most dominant, and were present in all symptomatic patients, supporting previous findings demonstrated elsewhere, such as an asymmetric peripheral neuropathy that is predominantly sensorial [12,13,14,16,28,29,30].

The most affected nerves were the ulnar in the elbow segment and the common fibular nerve in the fibular head segment, followed by the ulnar sensory nerves, superficial fibular, and sural. Although controversial, most of the researchers identify the ulnar as the nerve most compromised by leprosy, although any peripheral nerve can be affected by this neuropathy. The ulnar, median, common fibular, tibial, facial, cutaneous radial, and major auricular are the nerves most frequently reported in different studies [12,13,15,29,31,32]. This variability may be due to the different electroneuromyography protocols used, emphasizing the importance of an extensive and careful evaluation of leprosy neuropathy, mostly through an extended analysis of motor and sensory conductions, including nerves that are not evaluated in the routine.

The deep sensation and deep tendon reflex impairment, as well as the presence of muscular weakness and amyotrophy, generally occur in cases of prolonged evolution. In this study, motor impairment was more frequent in ulnar and fibular nerves. Apparently, the bacillus’s preference for colder areas of the body will lead to a focal reduction of the conduction velocity, which can be detected at some preferential sites, such as the ulnar nerve in the elbow area and the common fibular nerve in the fibula head area [13,16,33,34]

Neural thickening was present in the majority of the cases. However, the clinical evaluation of neural thickening is subjective, especially for professionals with limited experience, which may explain the very large data variation found among different examiners [35,36]. Besides that, the neural thickening is not a pathognomonic finding of leprosy neuropathy, which can be observed in compressive, inflammatory focal neuropathy, and even in hereditary neuropathies [13,14]. It should be emphasized that thickened nerves can also present a normal electroneuromyographic result [37], as observed in some of our patients.

The electroneuromyography of leprosy patients is very important, because it allows the stratification of the severity, the definition of patterns of the peripheral neural impairment, and also the early detection of PNL. These findings were reinforced by the large percentage of household contacts detected with this neuritic leprosy form, classified as oligo/asymptomatic patients, who do not always present neural thickening or other signs of this neuropathy.

In leprosy neuropathy, some patients seems to present a subclinical form, in which the sensory conduction study is superior to thermal sensation, vibratory, strength, and monofilament tests, with capability of detecting neural impairment in earlier phases [32,36]. Detection of patients with high disability level (grade 2) during diagnosis reinforces the complexity of these cases, leading to treatment delays in a clinical form whose neural damage is much more aggressive than the damage observed in other forms of the disease.

The absence or amplitude reduction of the sensorial action potential of ulnar nerves in leprosy patients can precede the disease’s classic clinical symptoms [38], which is corroborated by our electroneuromyography findings in oligosymptomatic patients, reinforcing the fact that the sensory ulnar nerve is the most biopsied nerve. Besides that, an early focal myelinic impairment of ulnar or fibular nerves was observed, also preceding the clinical manifestations of the disease [39]. However, there are doubts as to the electrophysiological changes most frequently found in this peripheral neuropathy, which reflects a disease with differential phenotypes that can evolve insidiously or through reactional episodes, accentuating neural damage and leading to functional disabilities and sequelae [40].

In relation to diagnostic tools, the diagnostic tests used for leprosy pursue direct demonstration of bacilli through biopsy and bacilloscopy, or indirect detection through serological or molecular analyses. These methods differ in sensitivity, specificity, reproducibility, and do not present a homogeneous response across leprosy forms; therefore, it is reasonable to investigate and adopt new methods to elucidate the neural forms [2].

The positivity of ELISA anti-PGLI during diagnosis in most cases emphasizes its importance as screening and as a risk indicator, particularly for oligo/asymptomatic patients. The anti-PGL-I seropositivity indicates that the bacillus has successfully penetrated the circulatory system, and represents a relative risk almost six times higher for disease occurrence, corroborating the leprosy neuropathy diagnosis. Nevertheless, the number of seropositive patients does not indicate existing infection prevalence, which reached 47.1% of our cases, since paucibacillary patients rarely produce specific antibodies [11,17,26,27,41].

The use of the polymerase chain reaction (PCR) to detect *M*. *leprae* DNA has been reported as a useful diagnostic tool for all clinical forms of leprosy, including the primary neural form, and is considered an important tool for early diagnosis [18,42,43]. Our study demonstrated a 78.6% positivity of *M*. *leprae* DNA in the slit skin smears, although all these patients were diagnosed with leprosy in their primary neural form, considering the current diagnostic criteria, which only require negativity in slit skin smear bacilloscopy. Therefore, it is important to emphasize that the concept of a pure neural form cannot be accepted, although it is often used as a synonym of the primary neural form, since only 21.4% of our cases presented a clinical and laboratory presentation that supports a uniquely neural involvement. In addition, clinical leprosy often starts from neurological symptoms instead of skin disease, and skin lesions may appear later [44].

The qPCR of peripheral nerves was positive in 60.8%, which has signficantly contributed to the leprosy diagnosis. There is a great variation in the literature related to the bacillus identification and to the histopathological analysis in peripheral nerve biopsy through conventional bacilloscopy, reinforcing the importance of qPCR use in these cases due to its greater sensitivity [16,18,45,46].

According to current recommendations for the operational classification of leprosy patients, which defines the multidrug therapy regimens, those cases with primary neural form that present more than one affected nerve, properly documented by loss or reduction of sensation in the respective areas, should be treated as a multibacillary form [20]. However, as observed in this study, many mononeuropathy patients presented high titers of anti-PGL-1 serology, which implies the patients’ bacillary load status and defines them as multibacillary cases with predominance of humoral immune response, considered a less aggressive disease, and also an early diagnosis, since other leprosy clinical signs were not present during diagnosis. If one considers only the number of affected nerves, treatment failures may occur, including disease relapse due to insufficient treatment.

Contrarily, lower anti-PGL-1 serology levels, characterizing the paucibacillary form, were observed in the multiple mononeuropathy patient group, suggesting a stronger cellular response and corroborating the greater immune aggression of nerves, which is evidenced by the greater severity of the peripheral neuropathy, with greater number of altered nerves, higher proportion of sensory-motor symptoms, and thickened nerves. Thus, diagnosis of both clinical and operational classifications of patients with the primary neural form still represents a big challenge.

Due to their hazardous and highly disabling neuropathy, we believe that all PNL cases should be treated with multibacillary treatment regimen, especially because of the lack of clear diagnostic criteria to distinguish paucibacillary from multibacillary patients, and also on account of the lack of association between the number of altered nerves and the bacillary load.

Early diagnosis of suspected leprosy neuropathy cases has been always posed a problem due to the long incubation period of the disease, the variable and insidious symptoms and clinical signs in both early and advanced cases, a context that reinforces the notion that PNL is underdiagnosed, causing severe disabilities and favoring the maintenance of the infection transmission chain.

**Fig 1:**
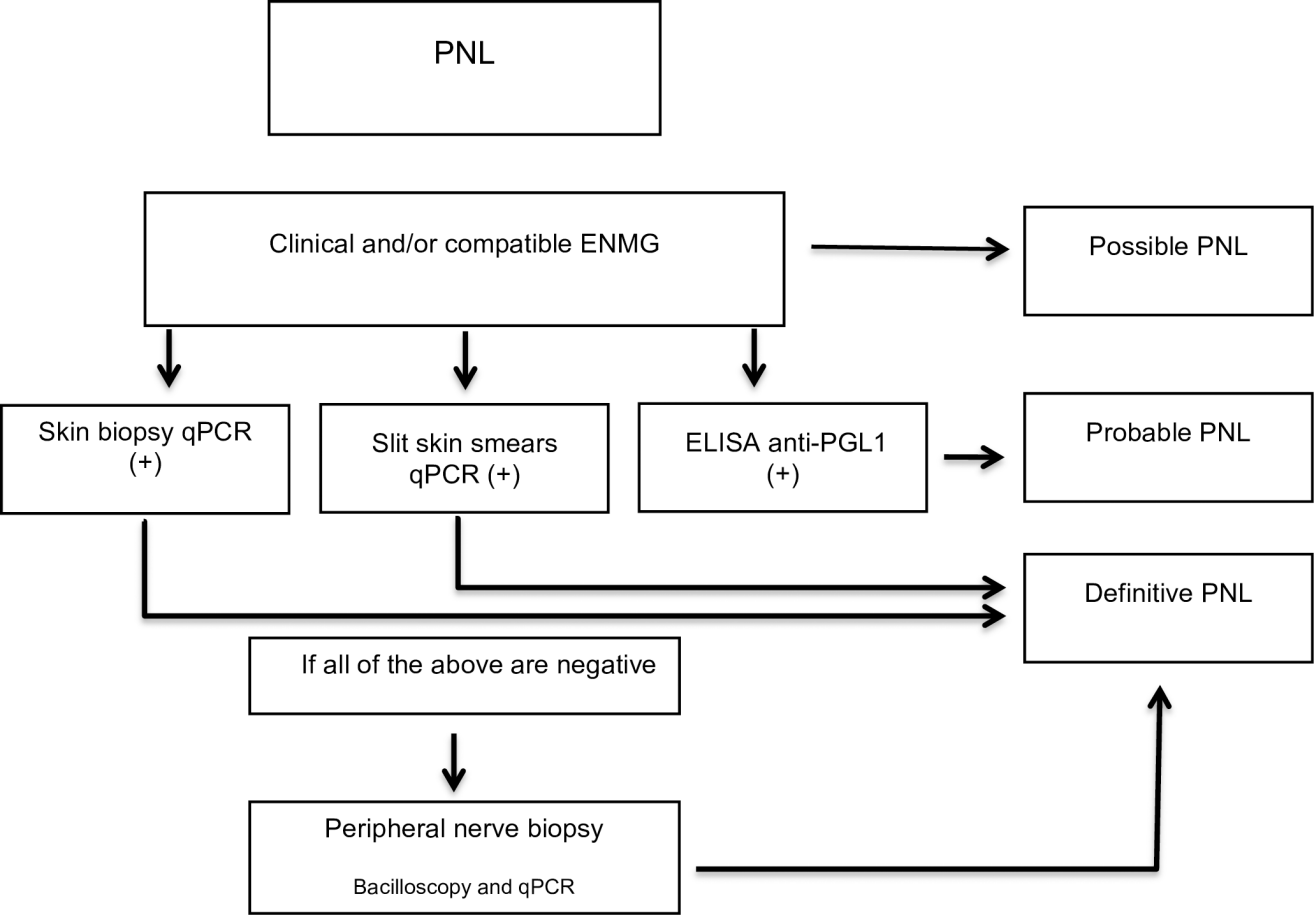
Algorithm and recommendations for PNL diagnosis. qPCR = Real Time Quantitative Polymerase Chain Reaction. ELISA = enzyme-linked immunosorbent assay. PNL = primary neural leprosy.

We propose an algorithm for PNL diagnosis (Fig 1) considering that for early recognition of the leprosy neural form, a severe public health problem in several developing countries and regions, the implementation of specific methods is an urgent requirement, including immunological, molecular and neurophysiological tools, which are necessary to elucidate the epidemiology of this disabling clinical form, thus contributing not only to an effective treatment, but also to the reduction of disabilities, deformities, and sequelae.

## Supporting information

S1 File Electroneuromyography. (DOCX)
